# Pediatric Invasive Aspergillosis

**DOI:** 10.3390/jof2020019

**Published:** 2016-06-13

**Authors:** Rachel L. Wattier, Lynn Ramirez-Avila

**Affiliations:** Department of Pediatrics, Division of Infectious Diseases and Global Health, University of California-San Francisco, 550 16th St, 4th Floor, Box 0434, San Francisco, CA 94143, USA; lynn.ramirez@ucsf.edu

**Keywords:** aspergillosis, pediatric, antifungal

## Abstract

Invasive aspergillosis (IA) is a disease of increasing importance in pediatrics due to growth of the immunocompromised populations at risk and improvements in long-term survival for many of these groups. While general principles of diagnosis and therapy apply similarly across the age spectrum, there are unique considerations for clinicians who care for children and adolescents with IA. This review will highlight important differences in the epidemiology, clinical manifestations, diagnosis, and therapy of pediatric IA.

## 1. Introduction

Invasive aspergillosis (IA) is one of the most common and serious infectious complications occurring in immunocompromised children. The incidence of IA appears to be increasing in children as it has in adults [1,2,3,4,5]. Significant advances have occurred in the diagnosis and therapy of IA in the last 15 years, and more recent studies indicate that clinical outcomes have improved during this time period [6,7]. However, treatment success and long-term survival after IA diagnosis remain suboptimal.

The evidence that informs care for children with IA is largely extrapolated from adult studies or based on small non-comparative pediatric studies, predominantly including children with malignancy and/or hematopoietic stem cell transplant (HSCT) recipients. This review will describe the known differences in epidemiology, clinical manifestations, diagnosis and therapy of pediatric IA, and will highlight areas of opportunity for further research.

## 2. Epidemiology

### 2.1. Incidence and Vulnerable Populations

The population-based incidence of IA in children is unknown and likely varies across healthcare settings and internationally. According to data from the Kids’ Inpatient Database, a representative dataset of inpatient hospitalizations in the United States, the annual incidence of IA was 437 cases/100,000 (0.4%) hospitalized immunocompromised children in the year 2000 [8]. Among the groups with highest incidence of IA are children severely immunocompromised due to allogeneic HSCT (annual incidence 4.5%), therapy for hematologic malignancies, especially acute myelogenous leukemia (AML; annual incidence 4%), and those with primary immunodeficiencies, particularly chronic granulomatous disease (CGD) (annual incidence 6.5%) [8]. Other populations susceptible to IA include children undergoing solid organ transplantation (SOT) (annual incidence 0.3%), autologous HSCT (annual incidence 0.3%), chemotherapy for solid tumor malignancy (annual incidence 0.1%), and those with advanced acquired immune deficiency syndrome or certain non-malignant hematologic disorders such as bone marrow failure syndromes or hemophagocytic lymphohistiocytosis [6,8,9,10,11]. Additionally, IA has been reported in children with systemic lupus erythematosus receiving high dose corticosteroids and other intensive immunosuppression, and has also rarely been reported in preterm neonates [12,13]. The major predisposing risk factors for IA are similar in adults and children (Table 1), except that risk factors within pediatric SOT recipients are poorly characterized due to limited studies.

### 2.2. Causative Species

The major causative species of IA are similar in children and adults, with multicenter studies reporting *Aspergillus fumigatus* as the most commonly identified species, responsible for 53% of cases in the largest pediatric series, followed by *A. flavus* [6,10,20]. Possibly due to differences in local epidemiology, some single centers have reported a predominance of *A. flavus* causing invasive disease in children [14,21,22]. This may also be due changes in clinical presentation of pediatric IA over time, since *A. flavus* is associated with primary cutaneous disease which was a common clinical presentation in early case series [23]. Other commonly reported species in children include *A. niger* and *A. terreus* [6,10,22].

### 2.3. Clinical Outcomes

Although estimates of mortality for children with IA are difficult to compare due to variable timing of outcome assessment, there have been apparent improvements in mortality over the past 15 years. A systematic review from 1995 to 1999 reported a 68% case-fatality rate for IA in patients under 20 years old [24]. Among 66 pediatric patients with cancer and IA diagnosed from 1962 to 1996, 85% died within one year following IA diagnosis [14]. In the largest multi-center study of children with IA, 52.5% of patients diagnosed between 2000 and 2005 died during therapy [10]. More recent pediatric cohort studies that have transitioned to earlier outcome assessments at 12 weeks following diagnosis report lower mortality rates of approximately 30% [5,6,25]. Long term survival of children with IA at a single oncology center improved from 12.5% during 1986–2000 to 58% during 2001–2010 [7,26]. A similar improvement in mortality has been observed in adults with IA [20]. Although this improvement is encouraging, 30% short-term mortality is suboptimal, and IA is associated with other adverse outcomes including prolonged hospitalization, increased hospitalization costs, and interruption in therapy for malignancy [5,8,15].

Risk for poor outcomes among children with IA is driven primarily by host immune status and burden of infection. Children who developed IA in the context of allogeneic HSCT in one study had 6-fold greater odds of death during therapy, compared to those with other underlying conditions [10]. Among children with IA in the context of malignancy, a longer duration of neutropenia following IA diagnosis is associated with increased mortality [27]. Disseminated *Aspergillus* infection has been associated with increased mortality, and non-pulmonary primary sites, such as cutaneous disease, have been associated with better outcomes [14]. Surgical treatment, though not consistently reported across studies, has been associated with improved survival [10].

## 3. Clinical Manifestations and Diagnosis

### 3.1. Clinical Manifestations

Similarly to adults, the lungs are the most commonly affected organ in pediatric IA [3,6,10,11,14,20,21,26,28,29,30,31]. In a review of 139 pediatric IA cases, 80% of children had pulmonary disease, 14% cutaneous, 13% sinus, 8% cerebral, and 4% tracheobronchitis [10]. Although earlier pediatric studies suggested that cutaneous IA is more common in children than adults [21], more recent studies report lower rates of 14%–20%; this is thought to be due in part to the increasing use of central over peripheral venous catheters in pediatric oncology patients [3,10,14]. Cutaneous IA can be primary from direct inoculation or secondary due to hematogenous dissemination from another site [32]. Several case reports describe primary cutaneous IA in preterm neonates [32,33,34].

Disseminated infection, defined as disease occurring in ≥2 sites, has been reported in 10.5% to 38% of children with IA and can be due to hematogenous or contiguous spread [3,7,10,14,21,28]. *Aspergillus* can be locally invasive, with spread from the lung to the pleura, chest wall, and heart, or from the sinus to the central nervous system (CNS). Although no known studies have been performed, some authors suggest screening children with pulmonary IA for CNS involvement given the high mortality, late appearance of symptoms, and impact on treatment [35]. Uncommon sites of IA include the eye [20], head and neck including otitis media and mastoiditis [26], thyroid [36], and gastrointestinal including liver [3,21,31], heart including endocarditis [37] and pericarditis [26], and bone [14,38].

Although some patients with IA present with isolated fever, the clinical manifestations vary depending on which organ system is involved [14]. Fever is common across all presentations. Patients with pulmonary IA can present with cough, dyspnea, hemoptysis/hemorrhage, hypoxia, and pleuritic symptoms [14,28,29]. Cutaneous IA manifestations can be nonspecific and include erythematous plaques, papules, nodules, pustules, blisters, ecchymosis, and eschar formation, often at sites of prior trauma including intravenous catheter and tape sites [14,21,32]. Facial pain, nasal congestion and discharge, visual changes, and headache have been reported in patients with sinus involvement [11,14,39]. CNS IA can present with changes in mental status, visual disturbances, seizure**s**, and focal neurologic deficits [14].

Atypical presentations without fever also occur [11]. It is also reported that up to 33% of patients with CGD who develop IA are asymptomatic and only 20% are febrile [30]. Patients with CGD, especially those infected with *A. nidulans,* can develop local extension to the pleura, chest wall, and vertebrae [30]. Unlike neutropenic patients who can suffer from extensive fungal angioinvasion with hematogenous dissemination, IA may cause more locally invasive disease in CGD patients due to an ongoing and ineffective neutrophilic inflammatory response with persistent hyphae [30]. Patients with hyper-IgE syndrome can present with fungal superinfection of pneumatoceles resulting in aspergillomas and subsequent risk for invasion to the lung parenchyma or disseminated disease [30].

### 3.2. Diagnostic Methods

Although prompt IA diagnosis is important given the high morbidity and mortality associated with this infection, existing modalities, including microbiology, pathology, fungal biomarkers, and radiology, have limitations. With currently available diagnostic methods, many children with suspected invasive mold infections do not have an identified pathogen, and are treated empirically [40]. The median time from onset of symptoms to diagnosis was 11 days (range 0–69 days) in one cohort, reflecting challenges in establishing a diagnosis with currently available methods [14]. Establishment of an optimal diagnostic approach is limited by few pediatric IA diagnostic studies, and inconsistent application of the European Organization for Research and Treatment of Cancer/Mycoses Study Group (EORTC/MSG) classifications of proven and probable IA across different studies [41,42]. Further studies in high-risk pediatric patients are needed to establish the ideal timing, frequency, and combination of tests that result in earlier diagnosis.

#### 3.2.1. Culture and Histopathology

Although culture from a sterile site and histopathologic evidence of tissue invasion remain the gold standard for diagnosis of IA, invasive procedures are needed to obtain samples and the diagnostic yield is low. In a pediatric cohort of 193 HSCT patients with fever, respiratory distress, or radiographic findings concerning for an invasive fungal infection (IFI), only 40% of the 101 bronchoalveolar lavages (BAL) performed were diagnostic, all for an infectious etiology, though 94% of the 19 lung biopsies performed yielded a diagnosis [43]. Furthermore, *Aspergillus* is rarely recovered from blood cultures [3,10,20]. Although the yield is low, positive cultures from affected sites are important because they identify the organism and provide antifungal susceptibilities in this era of emerging resistance even to first-line therapy [44]. Given the low yield of cultures and the morbidity and mortality of invasive procedures, the diagnosis of IA in many patients relies on indirect diagnostics including fungal markers and radiology.

#### 3.2.2. Galactomannan Antigen

Galactomannan is a polysaccharide in the *Aspergillus* cell wall that is released during fungal growth and can be measured using an enzyme immunoassay. The limited studies evaluating the use of serum galactomannan in children are heterogeneous; some of the studies are single center, focus on those with malignancies and HSCT, use different definitions for IA, antifungal prophylaxis regimens, and galactomannan positivity cut-offs [31,45,46,47,48,49,50,51,52]. Serum galactomannan for prospective screening of high risk patients and diagnosing IA has been studied mostly in immunocompromised adults [45,53,54,55,56]. The sensitivity and specificity of serum galactomannan for the diagnosis of IA in children is similar to adults (Table 2) [55,57]. Although earlier studies reported higher false positivity rates in children than adults [58], more recently, the false positive rate in a pediatric cohort was 5.2% [47]. Preterm neonates have a false positive galactomannan rate as high as 83% [59]. The cause of the high false positive rates in this population are not known but could be secondary to cross-reactivity with *Bifidobacterium spp*. that is a normal part of the neonatal GI flora and present in formula milk [59,60,61].

Although galactomannan increases with higher infectious burdens [70], it can have reduced sensitivity early in the infection and if the patient is receiving anti-mold therapy [70]. Lower galactomannan sensitivity and specificity has been reported in SOT patients [53]. Possibly due to the lack of angioinvasion, serum galactomannan has reduced sensitivity in patients with CGD [30,71]. In addition to screening and diagnosis, serum galactomannan has been studied as a predictor of clinical response and survival for patients with IA [72,73,74,75].

Galactomannan testing can also be performed on BAL fluid, cerebrospinal fluid (CSF), and urine. BAL galactomannan may be a helpful adjunct for the early diagnosis of IA though cut-off values for positivity have not been firmly established [69,76]. In a retrospective pediatric cohort of immunocompromised and immunocompetent patients, a BAL galactomannan cut-off of ≥0.98 optical density index (ODI) resulted in a sensitivity of proven or probable IA of 78% and specificity of 92% [69]. This BAL cutoff when combined with a serum galactomannan of ≥0.5 ODI yielded a sensitivity of 89% and specificity of 90% for proven or probable IA [69].

Few studies have assessed the utility of CSF galactomannan for the diagnosis of CNS IA and specific cut-off values for positivity have not been established. A recent study of pediatric and adult patients with CNS IA reported a CSF galactomannan sensitivity of 88% and specificity of 96% when using a cut-off of ≥1 ODI [77]. Urine galactomannan has also been studied in children though is associated with high false positive rates [47].

#### 3.2.3. Beta-d-Glucan

1,3-β-d-glucan (BDG) is a nonspecific cell wall component of many fungal pathogens including *Aspergillus*. A number of adult studies have assessed the performance of BDG in the diagnosis of IFI and specifically IA (Table 2) [62,78,79]. BDG for the diagnosis of IFI in children is limited to a few case reports and studies [46,67,80,81,82]. It has been difficult to establish an optimal positive cut-off for children because they have higher baseline levels than adults [83] and levels can be affected by colonization with *Candida* spp., among other causes of false positivity (Table 2) [84].

#### 3.2.4. Molecular Diagnostics

*Aspergillus* polymerase chain reaction (PCR) diagnostics are promising for pediatric IA. In a meta-analysis, the sensitivity of the blood *Aspergillus* PCR ranged from 84%–88% and the specificity from 75% to 76% [85]; the sensitivity of the BAL *Aspergillus* PCR ranged from 77% to 80% and specificity from 94% to 95% [85]. Fewer studies have assessed *Aspergillus* serum and BAL PCR for screening and diagnosis of IA in children [86,46,87,88,89,90]. Another emerging area is serum and BAL molecular diagnostics for identification of the *Aspergillus* species and the most common mutations that confer azole resistance [91,92]. *Aspergillus* PCR from the CSF [93], pleural fluid, fresh [94] and formalin-fixed paraffin-embedded tissue [95] has also been studied for the diagnosis of IA.

#### 3.2.5. Emerging Diagnostics

Given the limitations of established diagnostic methods, further investigation has attempted to identify additional non-invasive methods to diagnose IA in early stages. Different fungal organisms causing pulmonary infection are associated with characteristic profiles of volatile secondary metabolites that can be detected in exhaled breath [96]. A recent study showed 94% sensitivity (95% CI, 81%–98%) and 93% specificity (95% CI, 79%–98%) for a combination of exhaled metabolites to identify proven or probable pulmonary IA in a cohort of adults with suspected pulmonary IFI [96]. The lateral-flow device (LFD) is another promising test. This point of care test detects *Aspergillus* glycoprotein antigen in both serum and BAL has been studied in adult cohorts [97,98]. While these novel diagnostics shows promise, they have not yet been evaluated in children.

#### 3.2.6. Radiology

Plain film and computer tomography (CT) are used for the diagnosis of pulmonary IA, though radiographic findings overlap with those of other IFI. CT has higher sensitivity and specificity for early diagnosis of pulmonary IA [99,100]. Radiographic findings that are described as characteristic in adult pulmonary IA are less common in children. These include the ‘halo sign’ that occurs earlier in the course of infection, as well as cavitation and the ‘air crescent’ sign that occur upon immune reconstitution [10,101,102,103].

More commonly, pediatric patients with pulmonary IA have nodules, masses, peripheral infiltrates, consolidations, and pleural effusions [10,29,101,102,104]. In a retrospective review of pediatric patients with proven or probable pulmonary IA, the most common findings on CT or plain radiograph were nodules (59%) [10]. Nodules were more commonly reported in older compared to younger children (63% ≥13 year-olds *vs.* 72%, 6–12 year-olds *vs.* 39%, 0–5 year-olds). Cavities were reported in 25% of patients and a minority of patients had the halo (11%) and air crescent (2%) signs. The reasons for the lower rates of cavitation and the halo and air crescent signs in children are unknown but differences in pathophysiology, immune response, and timing of imaging are possible explanations [10,101,102,103].

Radiographic findings of sinus and CNS IA are nonspecific. Common radiographic findings of sinus IA are mucosal thickening (67%), opacification (48%), and air fluid levels (24%) [10]. Radiographic findings of CNS IA include hypoattenuating lesions on CT and T2 hyperintense lesions on MRI, single or multiple ring enhancing lesions, or dural enhancement abutting sinus disease [105,106].

## 4. Treatment

During the last 15 years, multiple new antifungals have become available for treatment of IA, expanding options within three different antifungal classes. However, licensing of new antifungals for use in children lags behind adult approvals, and there are important age-specific pharmacokinetic differences. Table 3 summarizes the antifungals with activity against *Aspergillus*, their current pediatric licensing status and evidence and/or consensus guidelines supporting their use in children with IA. Off-label use of medications is common in pediatrics and agents may be commonly used for age groups and/or indications that lack regulatory approval [6,10,107].

### 4.1. Amphotericin B Formulations (Polyenes)

Amphotericin B exerts fungicidal activity by binding to ergosterol and altering permeability of the fungal cell membrane. It is active against *Aspergillus* species except for *Aspergillus terreus* [140]. Conventional amphotericin B deoxycholate was the first available antifungal with activity against *Aspergillus*, and was for many years the only therapeutic option for IA. Its disadvantages are nephrotoxicity and infusion-related adverse effects. Lipid formulations of amphotericin B are better tolerated with less nephrotoxicity [111,112]. Though they differ from one another and from the parent drug in pharmacokinetics, there is no available evidence showing differences in efficacy between any of the formulations [141]. Differences in tissue distribution should be considered when treating infections at specific sites, such as the CNS, because tissue distribution varies between formulations [142]. Lipid amphotericin B formulations remain options for treatment of patients who are intolerant or unresponsive to voriconazole [138,139]. Their potential advantages include lack of drug interactions and broader spectrum of antifungal activity, for patients in whom IA is not confirmed and other fungi are suspected.

### 4.2. Triazoles

Triazoles inhibit biosynthesis of ergosterol in the fungal cell membrane, and are fungicidal against *Aspergillus* [143]. Itraconazole was the first available triazole with activity against *Aspergillus*, but its use for treatment of IA in children is limited due to unreliable bioavailability [120]. Voriconazole became the first line antifungal for IA based on a clinical trial demonstrating superior efficacy and tolerability of voriconazole compared to amphotericin B deoxycholate among patients 12 years and older with definite or probable IA [119,138]. Survival at 12 weeks of therapy was 70.8% in the voriconazole group compared to 57.9% in the amphotericin B group, and severe drug-related adverse events were less common with voriconazole [119]. Although there are no comparative trials in children with IA under 12 years, voriconazole is recommended as primary therapy for pediatric IA based on pediatric safety data and a non-comparative compassionate use study demonstrating complete or partial response to voriconazole in 45% of children with IA and other IFIs who were refractory to or intolerant of conventional therapy [118,139].

Substantially higher doses of voriconazole are needed in young children (<12 years) to achieve adequate drug exposure [113,114,115,116,117]. This is due to linear voriconazole elimination kinetics in children over a wider range of doses compared to adults and adolescents, who have non-linear elimination kinetics at usual doses [113]. Non-linear kinetics result in large increases in exposure with small dose increases, and can be observed in younger children receiving higher voriconazole doses. Additionally, the oral formulation of voriconazole has lower bioavailability in children (44%–65%) compared to adults (>90%); this may be due to more extensive intestinal first pass metabolism in children [114,115,144].

There is substantial inter-patient variability in voriconazole exposure, partly due to polymorphisms in CYP2C19, and this variability may be greater in children than in adults [113,114,115,116,145]. Therapeutic drug monitoring (TDM) of voriconazole is recommended with a target trough range of 1.0–5.0 mg/L [139]. Correlation of treatment failure with trough levels <1.0 mg/L has been shown in young children as well as adolescents and adults [146,147,148,149]. A recent meta-analysis showed clear associations between therapeutic voriconazole levels and treatment success, and between supra-therapeutic levels and toxicity [150]. Voriconazole TDM has demonstrated benefits in children and adults, by reducing avoidable discontinuation of voriconazole and increasing likelihood of a successful response to therapy [149,151].

The most common adverse events associated with voriconazole include hepatic abnormalities, visual changes, and rash, often attributable to photosensitization [113,115,116,118,119]. Less common but serious toxicities include neurotoxicity (confusion and/or hallucination) and prolongation of the QT interval with associated arrhythmias [116,119,152]. Children receiving voriconazole prophylaxis have developed severe phototoxic skin reactions; the mechanism of this reaction is unknown [153,154]. Long term voriconazole exposure has been associated with acceleration of chronic phototoxicity and increased risk for skin cancers in immunocompromised patients [155]. Long term use of voriconazole has also been associated with elevated serum fluoride levels, with associated skeletal fluorosis and painful osteitis [156,157]. Voriconazole is an inhibitor of CYP3A4, CYP2C19 and CYP2C9 and is therefore subject to multiple drug interactions.

Newer triazole agents, posaconazole and isavuconazole, may serve a greater role for therapy of pediatric IA in the future, but their use is currently limited by a paucity of pediatric data. Although there are no published clinical trials comparing efficacy of posaconazole against voriconazole for IA, it is recommended as an option for salvage therapy in patients ≥13 years of age on the basis of an externally controlled clinical trial in adults and adolescents with IA who were refractory to or intolerant of standard therapy [138,139,158]. It is available in oral suspension, oral tablet and IV formulations. Bioavailability of the oral suspension is unreliable and dependent on food intake [159]. This formulation has undergone limited evaluation in children, with observational studies characterizing pharmacokinetics, safety and efficacy in prophylaxis of invasive mycoses [122,123,124]. Preliminary results of a more extensive pediatric pharmacokinetic study indicate that target concentration attainment with the posaconazole oral suspension is poor, especially in young children [160]. The safety profile of posaconazole appears favorable, and further pharmacokinetic studies are in progress to establish optimal dosing of posaconazole in children, especially with the newer formulations, which are dosed differently than the suspension.

Isavuconazole, the most recently developed triazole, was found to be non-inferior to voriconazole for IA in adults [161]. Its potential advantages over voriconazole include broader spectrum of activity (including activity against mucormycosis), reliable pharmacokinetics, and a favorable safety profile [162]. However, there are currently no data to establish its use in children.

### 4.3. Echinocandins

Echinocandins inhibit synthesis of 1,3-β-d-glucan in the fungal cell wall and are fungistatic against *Aspergillus*. Caspofungin and micafungin are the best evaluated in children and have a favorable safety profile across multiple pediatric age groups [126,127,131,132,133]. Caspofungin and micafungin have been evaluated in non-comparative studies for treatment of patients with IA, including small numbers of children, mostly as salvage therapy for patients refractory to or intolerant of other agents [131,136]. Because of their fungistatic activity and lack of robust data for use as primary therapy, the echinocandins are recommended as second line therapy for IA in patients who are intolerant to or refractory to first line agents [138,139].

### 4.4. Combination Antifungal Therapy

Though not routinely recommended for primary IA therapy, use of combination antifungal therapy is common in both children and adults. Two recent multicenter pediatric cohort studies reported use of combination therapy in 46% and 54% of children with IA [6,10]. In one of these studies, children who received combination therapy were more likely to experience adverse events compared to those treated with monotherapy [6]. A recent clinical trial comparing voriconazole monotherapy to voriconazole and anidulafungin combination therapy for primary therapy of adults with IA showed a trend toward lower mortality with combination therapy (19.3% *vs.* 27.5% at 6 weeks; *p* = 0.087) but was underpowered for this primary endpoint [163]. The implications for management of children with IA are uncertain; recent European guidelines propose combination therapy as an option for pediatric IA, but with weak recommendations [139]. Combination therapy deserves further evaluation for safety and efficacy in children, given the major differences in antifungal pharmacology in children and adults.

### 4.5. Adjunctive Therapies

Colony stimulating factors and other therapies intended to reverse predisposing conditions and augment the immune response to *Aspergillus* are commonly given to children with IA [6,10,22]. As in adults, their specific contribution to clinical outcomes is uncertain, but an effect is biologically plausible due to the importance of immune reconstitution in recovery from IA. Adoptive transfer of donor-derived pathogen-specific T cells, a strategy used successfully against viral infections in HSCT recipients, is a promising option for IA therapy and is currently in early clinical development [164,165]. Many children with IA undergo surgery for disease control, with indications similar to those in adults; one pediatric study identified an association between surgical treatment and improved survival [10,22].

## 5. Conclusions

Important differences in diagnosis and treatment exist between adult and pediatric IA. Although there have been advances in the diagnosis, treatment, and outcomes of pediatric IA, challenges remain. Better characterization of IA in pediatric populations, such as those with SOT and primary immunodeficiencies, is needed. The greatest gains are likely to come from optimizing the diagnostic approach to IA, including incorporation of risk stratification, and establishing algorithms to determine which patients may be best evaluated through non-invasive tests, *versus* those that could benefit from invasive testing. Additional evaluation of combination antifungal therapy and pediatric studies of new antifungals will facilitate broader prophylaxis and treatment options in at-risk children. Novel treatment strategies such as immunotherapy may also play a role in the treatment of pediatric IA. Taken together, improved diagnostic and treatment modalities will enable earlier diagnosis and improve mortality for children with IA.

## Figures and Tables

**Table 1:** Identified Risk Factors for Invasive Aspergillosis in Children.

Risk Factor	Comments
**Shared by Multiple Groups**	
Prolonged and severe neutropenia [6,10,14]	
High dose corticosteroids [6,10,14]	
Other immunosuppression [6,10,14]	
**Specific to Children with Malignancy**	
Acute myelogenous leukemia (*vs.* other malignancies) [11,15]	Majority of episodes are diagnosed during intensive phases of therapy [15]
**Specific to HSCT ^a^ Recipients**	
Allogeneic transplant (*vs.* autologous) [8,10,11,16]	
Unrelated donor (*vs.* related donor) [10,11,16]	
Cord blood transplant (*vs.* other stem cell source) [16]	
Graft versus host disease [8,10,11,16]	
**Specific to SOT ^b^ Recipients**	
Lung transplantation (*vs.* other organ transplants) [4,8,10,11]	Other risk factors related to SOT are poorly characterized in children
**Specific to Primary Immunodeficiencies**	
Chronic granulomatous disease [17]	Higher risk in lower quartiles of superoxide production [17]
Hyper-IgE syndrome [18]	Risk associated with pneumatoceles following bacterial pneumonias [18]
Other severe defects of phagocyte and/or cellular immunity	e.g. Severe combined immunodeficiency, Wiskott-Aldrich syndrome [6,10,19]

^a^ Hematopoietic stem cell transplant; ^b^ Solid organ transplant.

**Table 2:** Fungal Biomarkers for Diagnosis of Invasive Aspergillosis.

Biomarker Attribute	Galactomannan Antigen (%, 95% CI)	1,3-β-d-glucan (%, 95% CI)
**Performance in Serum**		
Sensitivity—Adult ^a^ [55,62]	78% (70%–85%)	62% (48%–73%)
Sensitivity—Pediatric ^b^ [55]	84% (66%–93%)	Unknown
Specificity—Adult ^a^ [55,62]	85% (78%–91%)	91% (83%–95%)
Specificity—Pediatric ^b^ [55]	88% (60%–97%)	Unknown—likely less than adult
Causes of False Positive Result	Piperacillin-tazobactam, other beta lactam antibiotics, Plasmalyte, *Fusarium* spp., *Histoplasma capsulatum*, *Penicillium* spp. [45,60,63,64]	Ampicillin-sulbactam, piperacillin-tazobactam, intravenous immunoglobulin, albumin, certain hemodialysis filters, bacteremia [65,66,67]
**Performance in BAL ^c^ Fluid**		
Sensitivity—Adult ^d^ [68]	86%	n/a ^f^
Sensitivity—Pediatric ^e^ [69]	78%	n/a
Specificity—Adult ^d^ [68]	91%	n/a
Specificity—Pediatric ^e^ [69]	92%	n/a

^a^ Positivity cut-off ≥0.5 optical density index (ODI); ^b^ Subanalysis of 7 pediatric studies within a meta-analysis; 5 studies used a positivity cut-off ≥0.5 ODI, 1 a cut-off ≥1.5 ODI, one did not report cut-off; ^c^ BAL; bronchoalveolar lavage; ^d^ Positivity cut-off ≥0.8 ODI; ^e^ Positivity cut-off ≥0.98 ODI; ^f^ Not applicable, test not performed on BAL fluid.

**Table 3:** Antifungals for Treatment of Invasive Aspergillosis: Pediatric Licensing and Evidence Supporting Use in Children.

Class/Agent (Formulation—IV ^a^/PO ^b^)	Ages Currently Licensed ^c^	Pediatric Evidence	Clinical Guidelines
**Polyenes**			
Amphotericin B deoxycholate (IV)	All	First available agent	
Liposomal amphotericin B (IV)	≥1 month	PK ^d^, safety [108]. Observational cohort study [109]	IDSA ^d^: alternative primary therapy (A-I), salvage therapy (A-II). ECIL-4 ^e^: 1st line (B-I), 2nd line (B-II)
Amphotericin B lipid complex (IV)	All	Observational cohort study [110]. Non-comparative trial (salvage therapy) [111]	IDSA: alternative primary therapy (A-I), salvage therapy (A-II). ECIL-4: 1st line (B-II), 2nd line (B-II)
Amphotericin B colloidal dispersion (IV)	All (no longer commercially available)	Non-comparative trial (salvage therapy) [112]	IDSA: alternative primary therapy (A-I), salvage therapy (A-II)
**Triazoles**			
Voriconazole (IV, PO)	≥12 years ^g^	PK, safety [113,114,115,116,117]. Non-comparative trial (salvage therapy) [118]. RCT^f^ including children >12 years [119]	IDSA: primary therapy (A-I). ECIL-4: first line (A-I), second line for voriconazole-naïve patients (A-I)
Itraconazole (PO)	≥18 years	PK, safety [120]	IDSA: salvage therapy (B-II). ECIL-4: 2nd line (no grade)
Posaconazole (IV, PO)	PO: ≥13 years. IV: ≥18 years	PK, safety [121,122,123,124]. Retrospective cohort study [125]	IDSA: salvage therapy (B-II). ECIL-4: 2nd line (no grade)
Isavuconazole (IV, PO)	≥18 years		
**Echinocandins**			
Caspofungin (IV)	≥3 months	PK, safety [126,127,128,129]. Retrospective cohort study [130]. Non-comparative trial (primary & salvage therapy) [131]	IDSA: salvage therapy (B-II). ECIL-4: 2nd line (A-II)
Micafungin (IV)	≥4 months	PK, safety [132,133,134,135]. Non-comparative trial (salvage therapy) including pediatric patients [136]	IDSA: salvage therapy (B-II). ECIL-4: 2nd line (no grade)
Anidulafungin (IV)	≥18 years	PK, safety [137]	

^a^ IV; intravenous; ^b^ PO; oral; ^c^ Licensing status by United States Food and Drug Administration; not all drugs are licensed for primary therapy of invasive aspergillosis; voriconazole, isavuconazole, and amphotericin B deoxycholate are licensed for primary therapy; lipid amphotericin B formulations and caspofungin are licensed for salvage therapy in patients who are intolerant or unresponsive to other agents; posaconazole is licensed only for prophylaxis in the United States, but is licensed for salvage therapy by the European Medications Agency. Micafungin and anidulafungin are not licensed for IA but have *in vitro* activity similar to caspofungin.; ^d^ PK; pharmacokinetics; ^d^ IDSA; Infectious Diseases Society of America; combined adult and pediatric guidelines, evidence levels based on adult data [138]; ^e^ ECIL-4; Fourth European Conference on Infections in Leukemia; pediatric-specific guidelines [139]; ^f^ RCT; randomized controlled trial; ^g^ Voriconazole is approved by the European Medications Agency for children age 2–11 years.
